# Simultaneous Suppression of Multiple Programmed Cell Death Pathways by miRNA-105 in Cardiac Ischemic Injury

**DOI:** 10.1016/j.omtn.2018.12.015

**Published:** 2019-01-10

**Authors:** Sunhye Shin, Jung-Won Choi, Hanbyeol Moon, Chang Youn Lee, Jun-Hee Park, Jiyun Lee, Hyang-Hee Seo, Gyoonhee Han, Soyeon Lim, Seahyoung Lee, Sang Woo Kim, Ki-Chul Hwang

**Affiliations:** 1 Department of Integrated Omics for Biomedical Sciences, Graduate School, Yonsei University, Seoul 03722, Republic of Korea; 2 Institute for Bio-Medical Convergence, College of Medicine, Catholic Kwandong University, Gangneung-si, Gangwon-do 210-701, Republic of Korea; 3 Translational Research Center for Protein Function Control, Department of Biotechnology, Yonsei University, Seodaemun-gu, Seoul 03722, Republic of Korea; 4 Catholic Kwandong University, International St. Mary’s Hospital, Incheon Metropolitan City 404-834, Republic of Korea

**Keywords:** apoptosis, ischemic heart, microRNA-105, necroptosis, programmed cell death

## Abstract

Recent studies have shown that several upstream signaling elements of apoptosis and necroptosis are closely associated with acute injury in the heart. In our study, we observed that miR-105 was notably dysregulated in rat hearts with myocardial infarction (MI). Thus, the purpose of this study was to test the hypothesis that miR-105 participates in the regulation of RIP3/p-MLKL- and BNIP3-dependent necroptosis/apoptosis in H9c2 cells and MI rat hearts. Our results show that the RIP3/p-MLKL necroptotic pathway and BNIP3-dependent apoptosis signaling are enhanced in H9c2 cells under hypoxic conditions, whereas, compared with these pathways in the controls, those in miR-105-treated H9c2 cells are suppressed. Mechanistically, we identified miR-105 as the miRNA directly suppressing the expression of RIP3 and BNIP3, two important mediators involved in cell necroptosis and apoptosis. Furthermore, MI rat hearts injected with miR-105 had decreased infarct sizes, indicating that miR-105 is among three miRNAs that function simultaneously to suppress necroptotic/apoptotic cell death pathways and to inhibit MI-induced cardiomyocyte cell death at multiple levels. Taken together, miR-105 may constitute a new therapeutic strategy for cardioprotection in ischemic heart disease.

## Introduction

Heart disease, including heart failure, myocardial infarction (MI), and ischemia reperfusion (I/R), is one of the causes of mortality and morbidity worldwide.^1^, ^2^, ^3^, ^4^ Numerous studies have demonstrated that MI and I/R injuries lead to various types of cardiomyocyte cell death (e.g., necrosis, apoptosis, and autophagy).^5^, ^6^ For many years, apoptosis was considered the only form of regulated cell death, according to studies investigating myocyte cell death; apoptosis is a well-established programmed form of cell death that can be initiated by a mitochondria-mediated intrinsic pathway and death-receptor-mediated extrinsic pathway.^5^, ^7^ The programmed form of cell death has been described as an “unregulated” or “accidental” form of cell death. Many studies have focused on blocking apoptosis, because it was considered the only form of regulated cell death. However, recent advances have demonstrated that both apoptosis and necroptosis can be regulated in various types of cell death.^8^, ^9^, ^10^, ^11^ Moreover, necroptosis is another regulated cell death mechanism that exists in various diseases, including MI, I/R injury, heart failure, and inflammation,^11^, ^12^ and it is regulated by the necrosome, which consists of receptor-interacting protein kinase 3 (RIP3) and mixed lineage kinase-like (MLKL).^9^ Mechanistically, the initiation of necroptosis is triggered by death ligands, such as tumor necrosis factor (TNF)-α, TNF-related apoptosis inducing ligand (TRAIL), and pathogen-associated molecular patterns (PAMPs).^10^

In particular, RIP3 deficiency is a key determinant that protects the ischemic heart and improves cardiac function after ischemia- and oxidative-stress-induced myocardial necroptosis.^13^ Several studies have shown that RIP3 is involved in reactive oxygen species (ROS) production by mitochondria under high glucose-induced injury and inflammation in H9c2 cardiac cells.^14^, ^15^ Moreover, BCL2/adenovirus E1B 19-kDa protein-interacting protein 3 (BNIP3) is a well-known pro-apoptotic protein under hypoxic and ischemic conditions. BNIP3 promotes the apoptosis, necrosis, and autophagy of cardiomyocytes in disease states.^16^, ^17^, ^18^ Cardiomyocyte cell death due to high BNIP3 expression leads to mitochondrial permeability transition pore (MPTP) opening, mitochondrial swelling, and outer mitochondrial membrane (OMM) rupture by the release of cytochrome *c*.^16^ The disruption of BNIP3 inhibits apoptosis, is crucial for ischemic cardiomyocytes, and is important for cardiac cell survival.^16^, ^17^, ^18^, ^19^, ^20^ We also reported that downregulating BNIP3 is an effective means to prevent cardiac cell death.^21^ Nevertheless, inhibition of necroptosis or apoptosis remains limited as a therapeutic treatment in heart disease. However, recent studies have suggested a new strategy that combines targets of both necroptosis and apoptosis.^22^, ^23^

MicroRNAs (miRNAs)—endogenous, 22-nt-long, small non-coding RNAs—control gene expression by targeting mRNAs.^24^ Moreover, miRNAs regulate the expression of multiple genes by binding to target transcripts through imperfect sequence complementarity. The capacity of miRNAs to target multiple genes makes them useful therapeutic tools that can be more potent than agents that act on a single gene. miRNAs are known to play important roles in pathological conditions involving multiple cell death pathways, including MI and heart failure.^25^, ^26^ Recent studies have found that miRNAs regulate apoptosis, autophagy, and necroptosis by targeting key regulators under pathophysiological conditions.^27^, ^28^ Furthermore, synergistic interactions of miRNAs can increase the efficacy of therapeutics while reducing their side effects and slowing the development of drug resistance.^22^, ^29^, ^30^

In this report, we identified miR-105, a new miRNA that targets RIP3 and BNIP3. Moreover, we demonstrate that miR-105 activation may potentially offer a therapeutic approach to prevent myocardial cell death by inhibiting apoptosis and necroptosis in MI.

## Results

### Apoptosis and Necroptosis of Rat Heart following Myocardial Ischemia and MI

**Figure 1:**
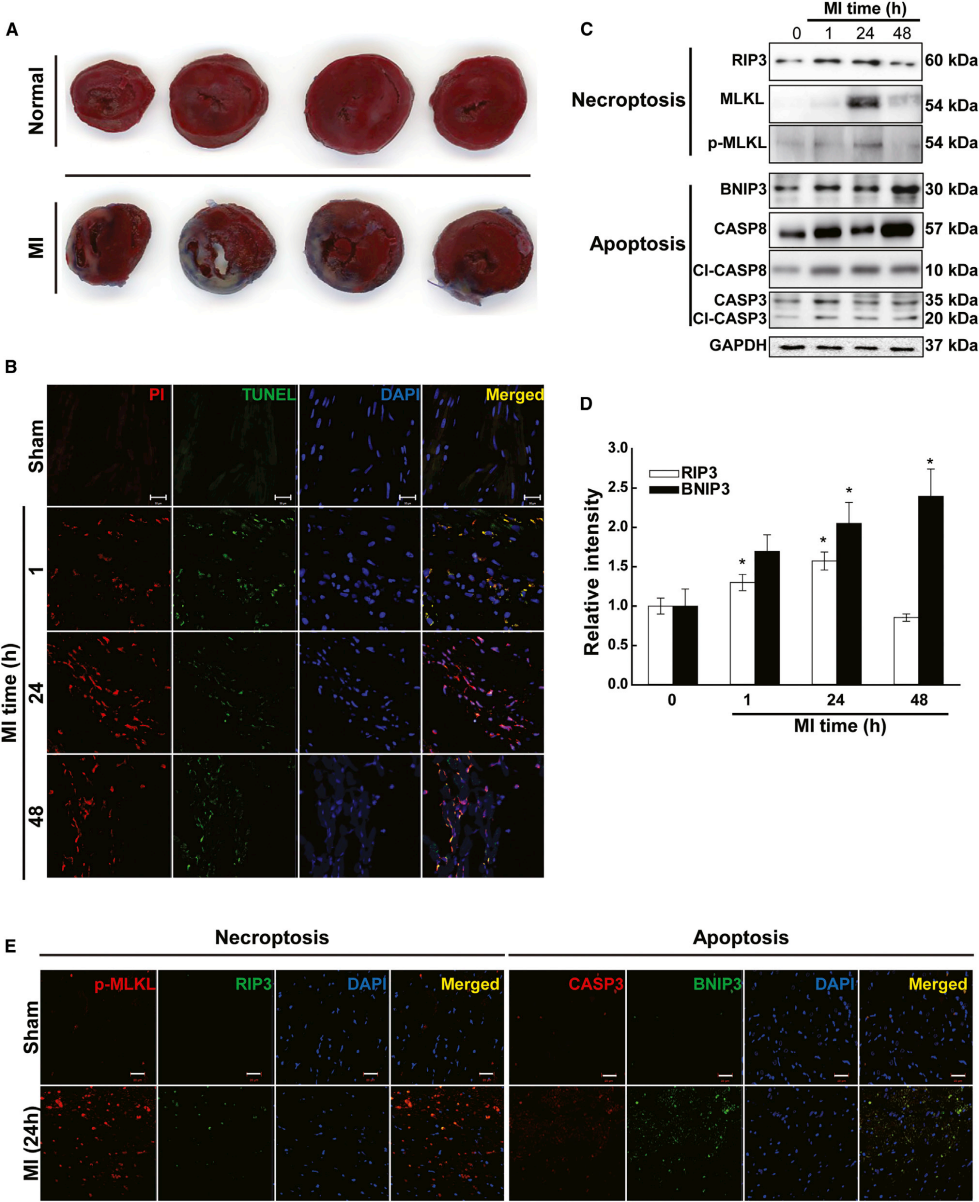
Activation of Apoptotic/Necroptotic Markers in MI Rat Hearts (A) TTC staining showing infarct areas in transverse sections. (B) Representative immunofluorescence images of staining with TUNEL (apoptotic cells) and PI (necroptotic cells) (n = 3). (C) Western blot bands showing apoptosis and necroptosis markers (n = 4). (D) Band intensities of RIP3 and BNIP3, which are important markers of apoptosis (BNIP3 and CASP3) and necroptosis (RIP3 and p-MLKL). The values given were normalized to the band intensity of GAPDH as an internal control. *p < 0.05; **p < 0.01; n = 4. (E) Representative immunofluorescence images of apoptosis and necroptosis marker staining in normal and MI rat hearts. Scale bars, 20 μm. n = 3.

First, we investigated the types of cell death distributed in the heart after MI (Figure 1). TTC (2,3,5-triphenyltetrazolium chloride) staining revealed that the hearts of healthy rats showed a uniform staining pattern, while MI-induced rats exhibited TTC-negative areas in the infarcted areas of the heart (Figure 1A). We used TUNEL and PI staining to examine the apoptosis and necroptosis levels in the heart following MI (Figure 1B). Compared with normal hearts, MI hearts showed higher levels of apoptosis and necroptosis. Western blot analysis showed that the levels of indicators of apoptosis (CASP3, caspase 8, and BNIP3) and necroptosis (RIP3 and p-MLKL) were significantly increased in hearts after MI (Figure 1C). Moreover, the highest level of BNIP3 was observed at 48 h, while the highest level of RIP3 was observed at 24 h after MI (Figure 1D). These data confirmed that apoptosis and necroptosis occurred in the heart after MI. Microscopic analysis of MI rat hearts showed that apoptosis markers (BNIP3 and CASP3) and necroptosis markers (RIP3 and p-MLKL) were found at higher levels in the infarcted areas of MI rat hearts (Figure 1E).

### Cell Viability and Key Signaling Molecule Expression Levels under Apoptotic and Necroptotic Conditions

**Figure 2:**
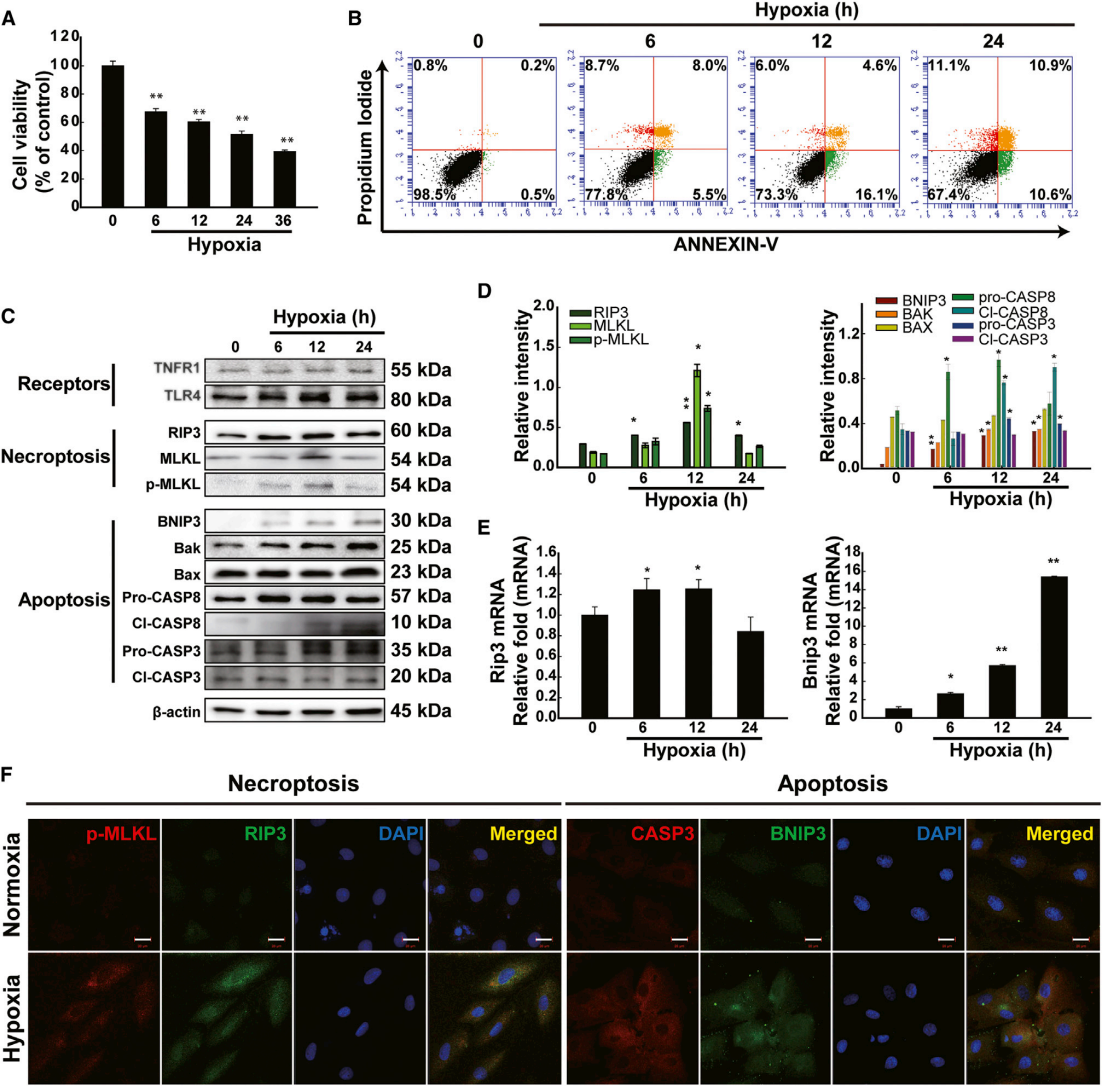
Apoptotic/Necroptotic Effects in H9c2 Cells under Hypoxic Stimulation (A) Cell viability against hypoxic stimulation (n = 3). (B) Flow cytometry analysis of apoptosis/necroptosis of H9c2 cells under hypoxic stimulation using Annexin V-PI (n = 3). (C) Differentiation of apoptosis/necroptosis markers and death receptors in response to hypoxic stimulation (n = 4). (D) Band intensities of apoptosis and necroptosis markers. The values given were normalized to the band intensity of β-actin as an internal control. *p < 0.05; **p < 0.01; n = 3. (E) Alteration of RIP3 and BNIP3 expression levels in response to hypoxic stimulation in H9c2 cells (n = 3). (F) Representative immunocytochemistry images of apoptosis (BNIP3 and CASP3) and necroptosis (RIP3 and p-MLKL) markers. Scale bars, 20 μm. The data are normalized to the 0-h control. The data are presented as the mean value ± SD of three separate experiments. n = 3.

To determine cell viability after hypoxia treatment, H9c2 cell viability was determined after various exposure times to hypoxia treatment conditions (Figure 2A). Significant decreases in cell viability were observed after 6, 12, 24, and 48 h of hypoxia treatment compared to that of the 0-h control sample under normoxic conditions (no hypoxic stimulation). Microscopic analysis of H9c2 cells compared to normoxic cells revealed decreased numbers and different morphology under hypoxic conditions (data not shown). We differentiated between two types of cell death (apoptosis and necroptosis) at the cell level by flow cytometry analysis with Annexin V-propidium iodide (PI) staining (Figure 2B). Double-negative cells indicate the live-cell population, Annexin V–/PI+ events represent apoptotic cells (10.6%), double-positive events represent dead cells (10.9%), and PI-positive/Annexin V-negative events represent necroptotic cells (11.1%) after 24 h of hypoxia. We further investigated relative changes in protein indicators of apoptosis (BNIP3, BAK, BAX, and CASP3) and necroptosis (RIP3 and p-MLKL) under hypoxia (Figures 2C and 2D). Consistent with the mRNA expression levels, the results of the western blot showed that apoptosis and necroptosis significantly increased after hypoxia and reached a maximum at 24 h and 12 h of hypoxia. Collectively, the results demonstrated that hypoxia induced apoptosis and necroptosis in H9c2 cells *in vitro*. Moreover, we examined effects of hypoxic stimulation on cardiac Toll-like receptor 4 (TLR4) and tumor necrosis factor receptor 1 (TNFR1) in H9c2 cardiomyocyte cells. The expression levels of the two receptors were increased after hypoxic treatment; the highest level of receptors was detected after 12 h of hypoxia in H9c2 cells. Apoptosis and necroptosis are induced by specific death receptors such as TNFR1 and TLR4, among other modules.^31^, ^32^ Under cell-death-inducing conditions, the TNFR1 complex is internalized and converted to a cell-death-inducing complex, termed complex II, with additional recruitment of Fas-associated protein with death domain (FADD) and caspase 8.^31^ TNFR1 signaling can also induce the necroptotic cell death complex, termed the necrosome, consisting of RIP1, RIP3, and FADD, which often occurs when apoptosis is blocked.^33^

To investigate the mRNA expression levels of RIP3 and BNIP3 under hypoxic conditions, H9c2 cells were exposed to varying lengths of hypoxia (0–24 h). Consistent with the MI heart results, the highest RIP3 mRNA level was detected after 12 h of hypoxia, whereas the mRNA levels of BNIP3 constantly increased with the duration of hypoxia (Figure 2E). BNIP3/CASP3 and RIP3/p-MLKL are widely used indicators to mark the cell death pathways of apoptosis and necroptosis, respectively. By immunohistochemical analysis, BNIP3/CASP3- and RIP3/p-MLKL-positive cells significantly increased after 12 h of hypoxia in H9c2 cells (Figure 2F). Many more cardiomyocytes undergoing apoptotic/necroptotic cell death appeared in hypoxia-treated cells than those in H9c2 cells in normoxic conditions.

### Identification of Specific miRNA Targeting RIP3/BNIP3

**Figure 3:**
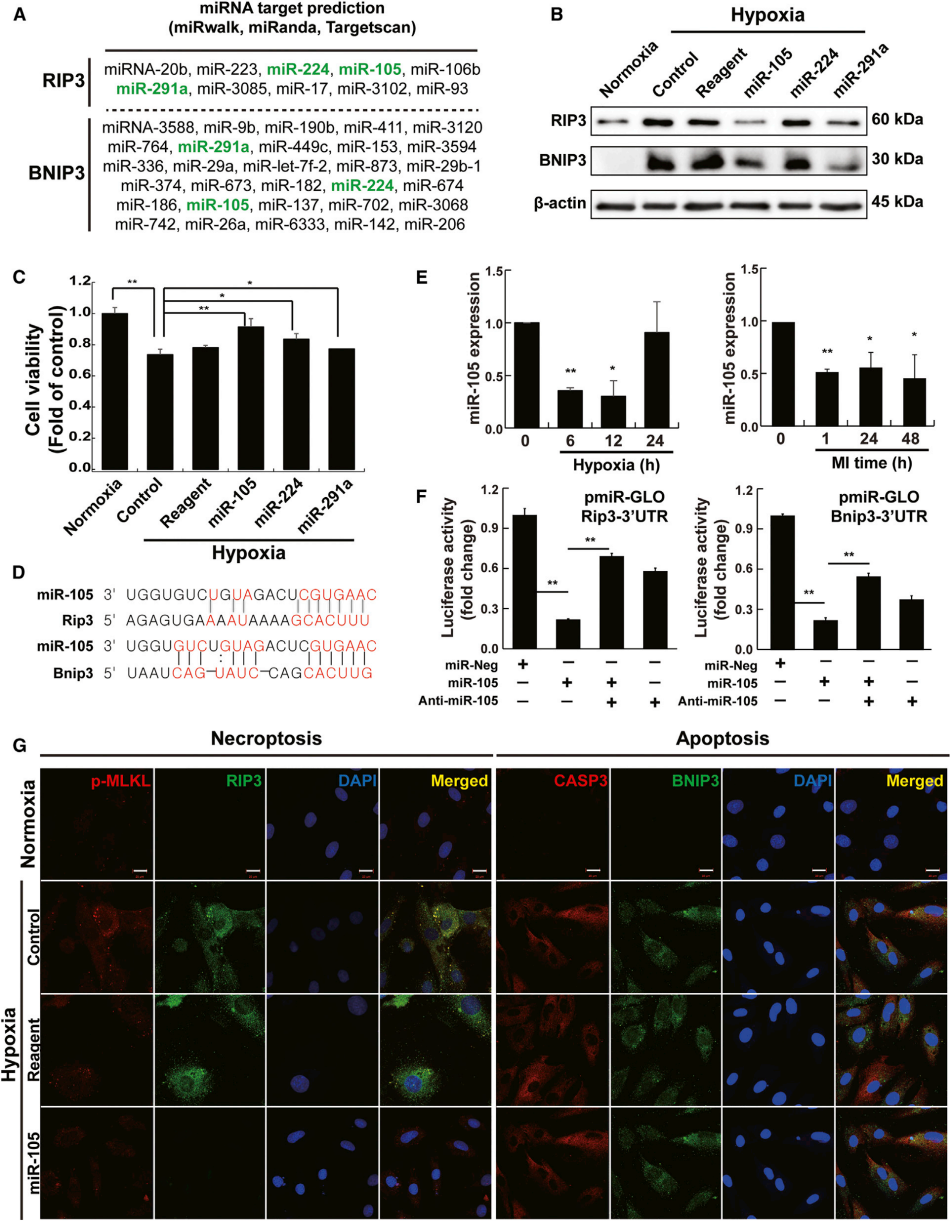
Identification of miRNAs Targeting RIP3 and BNIP3 to Suppress Multiple Programmed Cell Death Pathways (A) Candidate miRNAs were identified using three databases (TargetScan, miRwalk, and miRanda). (B) Effects of candidate miRNAs on RIP3 and BNIP3 expression levels upon hypoxic stimulation. β-actin was used as an internal control to normalize the expression of the target genes (n = 4). (C) Enhanced cell viability with candidate miRNA transfection upon hypoxic stimulation of H9c2 cells (n = 3). (D) Possible miR-105 binding site with Rip3/Bnip3. (E) Expression of miR-105 in hypoxia-stimulated H9c2 cells or in MI rat hearts (n = 3). (F) Luciferase assay using the 3′ UTRs of RIP3 and BNIP3. miR-Neg, negative control miRNA. *p < 0.05. The data are presented as the mean value ± SD of three separate experiments. (*p < 0.05; **p < 0.01; n = 3). (G) Representative immunocytochemistry images of miR-105 suppression effects on apoptosis and necroptosis upon hypoxic stimulation of H9c2 cells. Higher expression of BNIP3 and caspase-3 (CASP3) showing apoptotic cells in hypoxia-stimulated H9c2 cells, whereas expression was downregulated by miR-105. Higher expression of RIP3 and p-MLKL showing necroptotic cells in hypoxia-stimulated H9c2 cells, whereas expression was downregulated by miR-105. Scale bars, 20 μm. n = 3 independent experiments.

Candidate miRNAs (miR-105, miR-224, and miR-291a) controlling the multiple programmed cell death pathways were predicted and selected by their aggregate PCT scores, as assessed using miRNA target prediction databases (miRwalk, miRanda, and TargetScan) (Figure 3A). H9c2 cells transfected with miR-105 and -291a showed significant decreases in both RIP3 and BNIP3 protein expression levels compared to the control, while miR-224 showed no effects after 12 h of hypoxic conditions (Figure 3B). Consistent with the decreased RIP3 and BNIP3 protein expression levels, the highest cell viability was observed after 12 h of hypoxia in H9c2 cells transfected with miR-105 (Figure 3C). Cardiomyocyte cell death is associated with the multiple cell death signaling pathways, and other cell survival signaling pathways are likely associated with miR-291a modulation, but determining the exact cell survival mechanism is outside the scope of this study. Here, we attempted to identify miRNAs that can improve cardiomyocyte cell survival by reducing BNIP3/RIP3 levels, which, the results of the present study suggest, can reduce both apoptosis and necroptosis cell death signaling.

Possible association sites for miR-105 and Rip3/Bnip3 mRNAs are shown in Figure 3D. We confirmed the lower expression of miR-105 in hypoxia-treated H9c2 cells and MI rat hearts compared with that in control cells and hearts (Figure 3E). To further demonstrate that miR-105 targets the 3′ UTRs of RIP3 and BNIP3 mRNA, we utilized luciferase reporter constructs containing miR-105 binding sites of the RIP3 and BNIP3 3′ UTRs (Figure 3F). Transfection of miR-105 suppressed the expression of luciferase in cells with the miR-105 binding site but not in cells with the negative control miR-105 binding site. These data indicate that miR-105 targets the 3′ UTR regions of RIP3 and BNIP3 mRNA in a sequence-specific manner. As depicted in Figure 3G, miR-105 downregulated apoptosis indicators, such as BNIP3/CASP3, and necroptosis markers, such as RIP3/p-MLKL.

### miRNA-105 Suppresses Necroptosis and Apoptosis in Hypoxia-Treated H9c2 Cells

**Figure 4:**
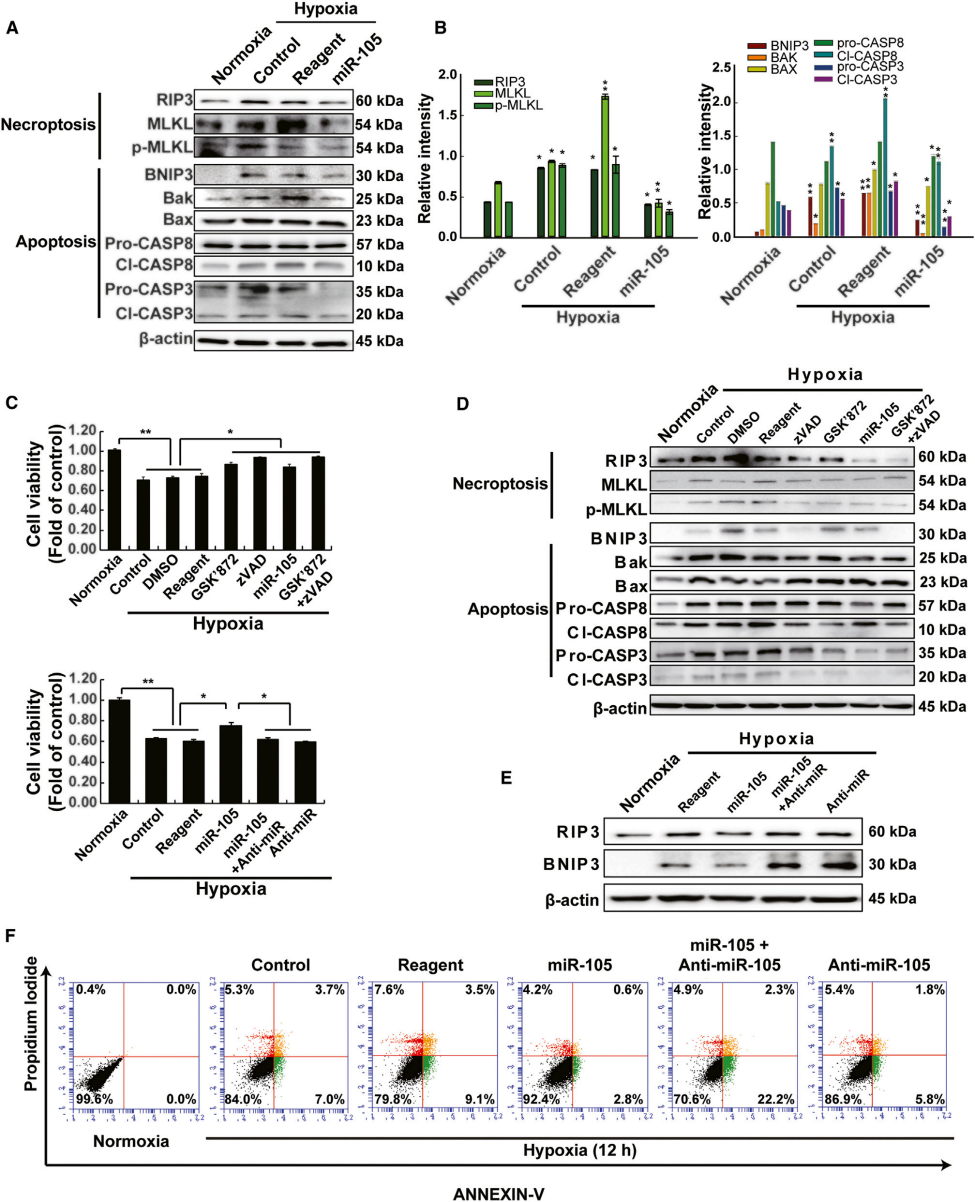
Simultaneous Suppression of Necroptotic and Apoptotic Cell Death by miR-105 Transfection in Hypoxia-Stimulated H9c2 Cells (A) Representative western blot bands showing apoptosis and necroptosis markers. n = 4. (B) Band intensities of apoptosis and necroptosis markers. The values given were normalized to the band intensity of β-actin as an internal control. *p < 0.05; **p < 0.01; n = 3. (C) Effects on cell viability by the inhibitor and anti-miR-105. n = 3. (D) Effect of necroptosis/apoptosis inhibitors and miR-105 against hypoxic stimulation in H9c2 cells. n = 4. (E) Verification of the efficacy and specificity of anti-miR-105 in silencing miR-105 at the protein level. n = 4. (F) Anti-necroptotic/anti-apoptotic effects of miR-105 under hypoxic conditions in H9c2 cells by flow cytometry analysis using Annexin V-PI. n = 3.

To examine the role of miR-105 in the regulation of apoptosis and necroptosis, cell cytotoxicity was examined in hypoxia-treated H9c2 cells; miR-105 treatment suppressed BNIP3/RIP3 in the cells (Figures 4A and 4B). In addition, the role of miR-105 in the regulation of apoptosis and necroptosis was verified in hypoxia-treated primary cardiomyocytes (Figure S1). To evaluate the relative contributions of necroptosis/apoptosis to hypoxia-induced cell death, we used a CCK assay, which measures both apoptotic and necroptotic cell death, in the presence or absence of the pan-caspase inhibitor zVAD, the RIP3 inhibitor GSK’872, and anti-miR-105 (Figure 4C). Hypoxia-induced cell death could be blocked by zVAD or GSK’872 alone, suggesting that, under the conditions used, hypoxia-induced cardiomyocyte death is mediated by both cell death pathways. Notably, miR-105-transfected cells exhibited the highest cell survival efficiency as in the zVAD-GSK’872 combined treatment. Moreover, BNIP3 and RIP3 expression decreased more in miR-105-transfected cells compared with that in the cells subjected to combined zVAD-GSK’872 treatment (Figure 4D). We next determined the functional role of miR-105 with anti-miR-105 against hypoxic stimulation in H9c2 cells (Figure 4E). The results suggest that BNIP3 and RIP3 expression is specifically downregulated by miR-105 treatment in hypoxia-stimulated H9c2 cells. To determine whether transfection affects cell viability, we examined two types of cell death (apoptosis and necroptosis) by flow cytometry analysis with Annexin V-PI staining under hypoxic conditions (Figure 4F). Annexin V–/PI+ events indicate apoptotic cells (9.1%), double-positive events represent dead cells (3.5%), and PI-positive/Annexin V-negative events represent necroptotic cells (7.6%) after 12 h of hypoxia. As expected, miR-105-transfected H9c2 cells exhibited enhanced cell viability against hypoxia-induced apoptosis/necroptosis. The number of apoptotic and necroptotic cells was markedly decreased in the presence of miR-105. Moreover, in accordance with our *in vitro* experiments under hypoxic conditions, we confirmed that miR-105 was significantly downregulated in MI rat hearts.

### miRNA-105 Suppresses Necroptosis/Apoptosis in MI Rat Hearts

**Figure 5:**
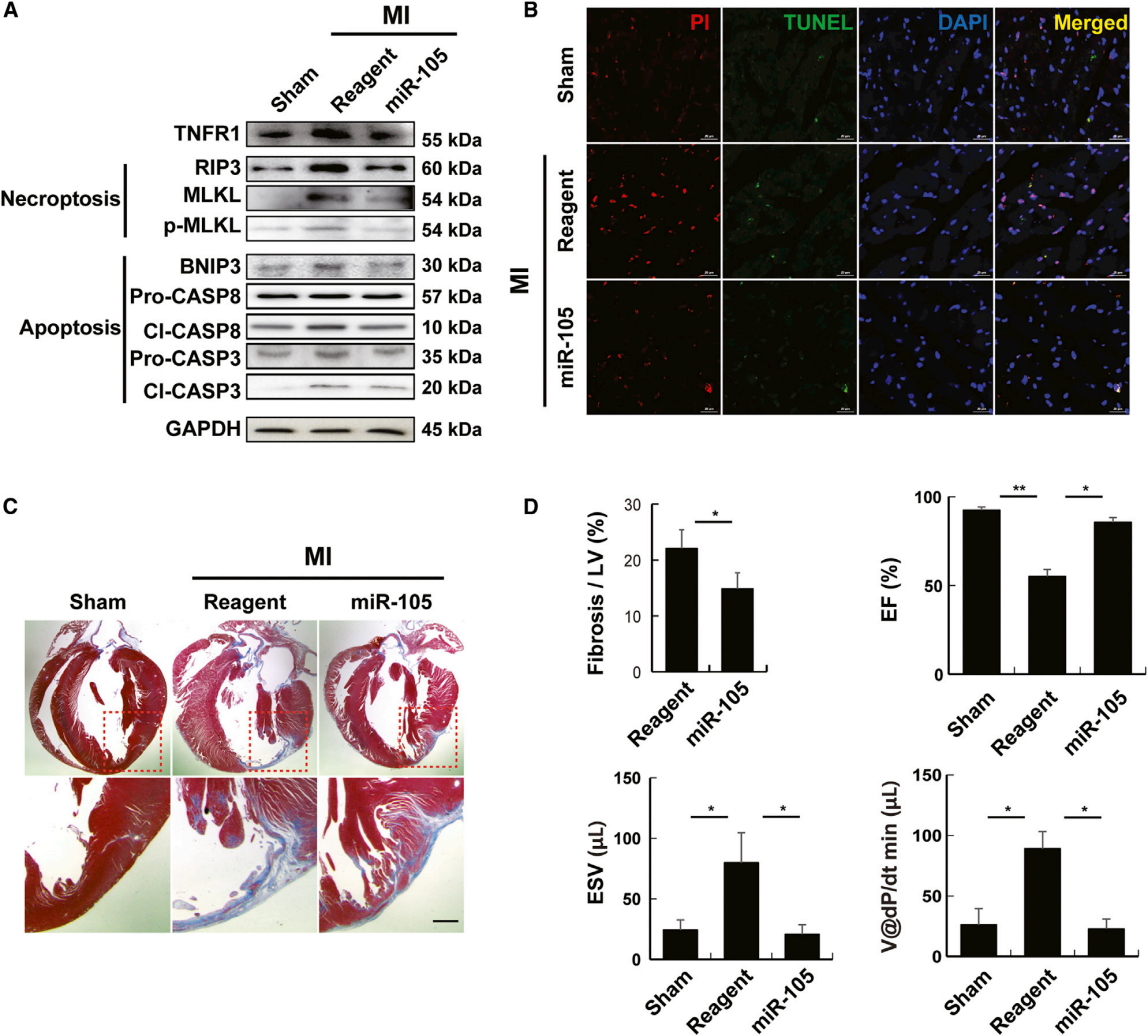
Anti-necroptotic/Anti-apoptotic Functions of miR-105 in MI Rat Hearts (A) Representative western blot bands showing apoptosis and necroptosis markers. GAPDH was used as an internal control to normalize the expression of the target genes. n = 4. (B) Representative immunofluorescence images of staining with TUNEL (apoptotic cells), PI (necroptotic cells), and DAPI. Scale bars, 200 μm. n = 3. (C) Histological analysis of MI rat hearts after miR-105 injection. Cardiac fibrosis was evaluated by Masson’s trichrome staining. n = 3. (D) Cardiac function analysis. EF, ejection fraction; ESV, end-systolic volume; V@dP/dt min, volume at dP/dt min. n = 3 independent experiments.

We tried to clarify whether the anti-necroptosis/anti-apoptosis effects of miR-105 observed in H9c2 cells under hypoxic conditions also exist in *in vivo* in MI rat hearts (Figure 5). Western blot data showed that, compared to the control MI rat hearts, the MI rat hearts transfected with miR-105 showed significant decreases in both RIP3 and BNIP3 protein expression levels (Figure 5A). Consistent with the *in vitro* results, TUNEL and PI staining analysis showed that cardiomyocyte necroptotic/apoptotic cell death induced by MI was markedly reduced in miR-105-treated rat hearts (Figure 5B). MI rat heart tissue showed significantly increased cardiomyocyte necroptosis/apoptosis, and treatment with miR-105 drastically decreased this ischemic necroptosis/apoptosis compared with that in MI rat hearts. In conclusion, miR-105 synergistically inhibits RIP3 and BNIP3 against myocardial cell death. Furthermore, we determined the functional role of miR-105 in infarcted hearts and found that miR-105 significantly reduced the infarct size in MI (Figure 5C). Trichrome staining of the heart demonstrated that miR-105 significantly attenuated cardiac fibrosis. In addition, cardiac function parameters, including the ejection fraction (EF), end-systolic volume (ESV), and volume at dP/dt min (V@dP/dt min) were significantly improved by miR-105, compared to those in the MI rat hearts (Figure 5D). Altogether, based on these *in vivo* and *in vitro* data, we conclude that both cardiomyocyte necroptosis and apoptosis have important roles in hypoxia-induced myocardial injury. miR-105 functions to simultaneously suppress necroptotic/apoptotic cell death pathways and cooperatively inhibit MI-induced cardiomyocyte cell death.

## Discussion

In this study, we observed that miR-105, which targets RIP3/BNIP3, was notably dysregulated in rat hearts with MI. The purpose of this study was to test the hypothesis of whether miR-105 participates in the regulation of RIP3/p-MLKL- and BNIP3-dependent cell death pathways, necroptosis and apoptosis, in H9c2 cells and MI rat hearts.

miRNAs are involved in regulating myocardial injuries and cardiac functions in the setting of acute MI (AMI).^29^, ^34^, ^35^ Furthermore, miRNAs play important roles in pathological conditions involving apoptosis, including AMI and heart failure.^33^ Apoptosis has been considered a possible target for novel therapies in heart failure, as this process is tightly regulated by specific signaling pathways and could thus potentially be inhibited.^36^ However, the overall rate of apoptotic cells in the infarcted region was <1% in a recent study, and recent theories have questioned the significance of the role of apoptosis in post-ischemic remodeling. In recent years, necroptosis has been described as another regulated cell death form that exists in various diseases, including MI.^37^ However, whether all cell death mechanisms in MI affect subsequent cardiac remodeling processes remains largely unknown. Recent studies have suggested that necroptosis inhibition is involved in cardioprotection of ischemic preconditioning and is associated with inhibition of the translocation of MLKL within the plasma membrane.^38^ Very recently, it was shown that Ripk3 promotes endoplasmic reticulum (ER) stress-induced necroptosis via the calcium overload/XO/ROS/mPTP opening axis in cardiac IR injury.^33^

A recent study reported that the combination of two miRNAs (miR-21 and miR-146a) synergistically decreased apoptosis under ischemic/hypoxic conditions in acute MI in mice.^29^ Among the cell-death-pathway-regulating miRNAs, both miR-21 and miR-146a have been documented to elicit anti-apoptotic effects and, thereby, beneficial effects on ischemic myocardial injury.^29^ As further evidence, miR-98 overexpression attenuated the upregulation of Fas and caspase-3 in H_2_O_2_-treated cardiomyocytes at the mRNA and protein levels.^39^ Furthermore, MI mice injected with miR-98-agomir had a significant reduction in the number of apoptotic cells, serum LDH levels, myocardial caspase-3 activity, and Fas and CASP3 expression in heart tissues. Similarly, we previously demonstrated that miR-223-5p and -3p cooperatively suppress necroptosis in ischemic/reperfused hearts.^40^ MicroRNA-103/107 regulates programmed necrosis and myocardial ischemia/reperfusion injury by targeting FADD.^41^ The long noncoding RNA H19 directly binds to miR-103/107 and regulates FADD expression and necrosis. Another investigation suggested that miR-894 regulates myocardial necrosis by targeting caspase 8,^42^ and Foxo3a can transcriptionally repress miR-894 expression, implying that modulation of Foxo3a, miR-874, and caspase 8 levels may provide a new approach for tackling myocardial necrosis.

However, for the first time, our study confirmed that miR-105, which targets RIP3/BNIP3, simultaneously dysregulates necroptotic and apoptotic cell death in rat MI hearts and H9c2 cells under hypoxic conditions. We identified the signaling pathway responsible for the anti-necroptotic/apoptotic effects of miR-105 against hypoxia-induced myocardial injury *in vivo* and *in vitro*.

A large amount of evidence has placed RIP3/MLKL and BNIP3/CASP3 in a central position in the pathogenesis of ischemia- and oxidative-stress-induced cardiac injury and heart failure.^29^, ^37^, ^39^, ^43^ For many years, apoptosis was considered the only form of regulated cell death, and studies investigating MI mainly focused on apoptosis.^44^, ^45^ In recent years, necroptosis has been found to be another regulated cell death type existing in various diseases, including MI;^46^, ^47^, ^48^ however, few studies have focused on necroptosis in MI.^37^ However, whether all mechanisms of cell death in MI affect the subsequent cardiac repair process remains largely unknown. In this study, we observed that cardiomyocyte cell death upon hypoxic treatment was attenuated significantly when treated with GSK’872 (a RIP3 inhibitor) or zVAD (a caspase inhibitor). Moreover, miR-105 significantly ameliorated cell injury and attenuated cell death, as did the combined zVAD-GSK’872 treatment against hypoxic conditions in cardiomyocytes. In agreement with the *in vitro* results, we found that miR-105 functions to simultaneously suppress BNIP3/RIP3 expression levels, which are the primary mediators of necroptotic/apoptotic cell death pathways in MI rat hearts. However, we did not precisely examine whether the multiple programmed cell death pathways under hypoxic conditions were necroptotic/apoptotic cell death, which represents a limitation of this study, particularly for elucidating the underlying mechanisms of miRNA-mediated cardiomyocyte cell survival effects. Further studies using necroptosis and apoptosis inhibitors specific for necroptosis and apoptosis factors will be helpful in identifying the exact cell death type.

In conclusion, the present study shows that the distribution of apoptosis and necroptosis differs in a time-dependent manner after MI or hypoxia stimulation. Moreover, miR-105 treatment improves the cell viability of H9c2 cells by inhibiting apoptosis and necroptosis, which is associated with the regulation of the interplay between them. Thus, our results offer strategies for the treatment of MI and post-MI cardiac remodeling.

## Materials and Methods

### Materials

H9c2 cells were obtained from the Seoul Korean Cell Line Bank (Seoul, Korea). DMEM and penicillin-streptomycin were obtained from Thermo Fisher Scientific (Waltham, MA, USA). Fetal bovine serum (FBS) was obtained from Atlas Biologicals (Atlas Biologicals, Fort Collins, CO, USA) for H9c2 cell culture. The following primary antibodies were used for immunoblot assays: BNIP3, BAK, BAX, CASP3, caspase 8, MLKL, p-MLKL, and RIP3. Horseradish peroxidase (HRP)-conjugated anti-mouse immunoglobulin G (IgG), anti-goat IgG, and anti-rabbit IgG were obtained from Santa Cruz Biotechnology. Detailed information of the antibodies is listed in Table S1.

### Animal Experiments and Histological Analysis

All experimental procedures for animal studies were approved by the Committee for the Care and Use of Laboratory Animals of Catholic Kwandong University College of Medicine (CKU01-2017-002) and were performed in accordance with the Committee’s Guidelines and Regulations for Animal Care. Seven-week-old male Sprague-Dawley rats (220 ± 30 g; n = 10 per group) were used for the MI model and were anesthetized via intraperitoneal injection of tiletamine/zolazepam (Zoletile, 30 mg/kg) and xylazine (10 mg/kg). The rats were ventilated via the trachea using a ventilator (Harvard Apparatus, Holliston, MA, USA) and then subjected to median sternotomy. MI was induced by tightened ligation of the left anterior descending coronary artery using a 7-0 Prolene suture (Covidien, Dublin, Ireland) for 1, 24, or 48 h. To obtain cardiac tissue, we perfused hearts with PBS and fixed them with 4% formaldehyde.

### Infarction Size Analysis and Cell Death Assays of Heart Tissues

To measure myocardial infarct size, 2–3 mL 2% Evans blue solution (Sigma, St. Louis, MO, USA) was transcardially perfused. The heart was subsequently removed and washed with saline. Prior to being divided into six 2- to 3-mm sections, the hearts were perfused with 1% TTC (Sigma, St. Louis, MO, USA) for 1 h at 37°C and incubated in 4% formaldehyde overnight at 4°C. The heart sections were photographed with a digital camera. The area at risk (red staining) indicating the ischemic area, the infarcted area (white staining), and the non-ischemic area (blue staining) were observed. The infarcted area was measured directly by planimetry of normal and infarcted left ventricular myocardia using ImageJ software. In infarcted hearts, apoptotic and necrotic cells were determined using PI and TUNEL staining. All PI-positive cells indicated necrotic cell death, and cells that were TUNEL positive only indicated apoptotic cell death. In brief, sections of heart tissue were incubated with PI without permeabilization, and then TUNEL staining was performed per the manufacturer’s instructions. The nuclei were stained with DAPI and examined under virtual microscopy (BX51 Dot Slide; Olympus, Tokyo, Japan).

### Cell Culture and Induction of Hypoxia

The rat cardiomyocyte-derived H9c2 cell line (American Type Culture Collection) was cultured in high glucose-DMEM (GIBCO, Waltham, MA, USA) containing 10% FBS (Atlas Biologicals, Fort Collins, CO, USA) and 1% antibiotics (GIBCO). To induce necroptosis and apoptosis, cells were incubated in a hypoxic chamber (Thermo Fisher Scientific) with 1% O_2_, 5% CO_2_, and 94% N_2_. After exposure to hypoxia, cell counting kit solution (CCK-8, Dogen, Seoul, Korea) was added to each well at a final concentration of 0.5 mg/mL and incubated for 2 h at 37°C. Cell viability was determined by measuring cells at 450 nm.

### Transfection of miRNA and Anti-miRNA

Transfection of miRNA (Genolution Pharmaceuticals, Seoul, Korea) was performed using the TransIT-X2 system (Mirus Bio, Madison, WI, USA) for 12 h. The miRNA-105 sequence is 5′-CAAGUGCUCAGAUGUCUGUGGU-3′. H9c2 cells were transfected with a final concentration of 10 nM miRNA according to the manufacturer’s instructions. Anti-miRNA-105 (10 nM) was used to inhibit the expression of endogenous miRNA-105. After transfection of miRNA or anti-miRNA, the media were changed for stabilization, and then hypoxia was induced.

### Real-Time RT-PCR

Total RNA from cultured cells and heart tissues was extracted using TRIzol reagent (Life Technologies, Frederick, MD, USA), and cDNA was synthesized using the RT PreMix kit (Bioneer, Daejeon, Korea). The level of each gene transcript was quantitatively determined by qPCR using the StepOnePlus Real-Time PCR System (Applied Biosystems, Foster City, CA, USA) with the SYBR Green dye system (SYBR Premix Ex Taq [Tli RNase H Plus, ROX Plus], Takara Bio, Foster City, CA, USA). The transcript level of each gene was normalized to GAPDH transcript levels. The level of miRNA transcripts was quantitatively determined using reverse transcription (TaqMan MicroRNA Reverse Transcription Kit; Applied Biosystems, Waltham, MA, USA) in combination with TaqMan miRNA assays, assays for quantification of miRNAs (miR-105, miR-224, and miR-291a), and U6 control transcripts according to the manufacturer’s instructions. The threshold cycle (Ct) values of miRNAs and U6 expression levels were automatically defined, located in the linear amplification phase of the PCR, and normalized to the control U6 (ΔCt value). The relative differences in the expression levels of miRNAs in the sorted cells (ΔΔCt) were calculated and presented as the fold induction (2^−ΔΔCt^). Detailed information about the primers is listed in Table S1.

### Immunoblot Analysis

Cells were lysed with RIPA buffer (Thermo Fisher Scientific) containing 1% phosphatase inhibitor and 1% protease inhibitor. Protein concentrations were determined using the Pierce BCA Protein Assay Kit (Thermo Fisher Scientific), and 10 μg protein was diluted in sample buffer (50 mM Tris [pH 6.8], 2% SDS, 10% glycerol, 0.1% bromophenol blue, and 5% β-mercaptoethanol) and heated for 5 min at 99°C. Next, proteins were separated by SDS-PAGE and transferred to a polyvinylidene difluoride (PVDF; Millipore) membrane. The membrane was blocked for 1 h with 5% skim milk in TBS (Tris-buffered saline)-T buffer (containing 10 mM Tris-HCl, 150 mM NaCl, 0.1% Tween 20) and incubated overnight at 4°C with a primary antibody. Primary antibody was diluted in TBS-T buffer containing 5% BSA (AMRESCO, Solon, OH, USA) and 0.02% sodium azide (Sigma-Aldrich). After five washes, the membrane was incubated for 1 h with HRP-conjugated anti-mouse IgG, anti-goat IgG, or anti-rabbit IgG (1:2,000, Santa Cruz Biotechnology) in blocking buffer and then washed five times. The membranes were visualized using an enhanced chemiluminescence system (ECL; Western Blotting Detection Kit, GE Healthcare), and the band intensities were quantified using ImageJ software.

### Multicolor Immunofluorescence Staining

For analysis of target protein expression patterns in rat hearts under pathological conditions, perfused hearts were fixed with 4% formaldehyde and embedded in paraffin. Three-micron-thick sections were mounted on gelatin-coated glass slides. After deparaffinization and washing with PBS, sections were incubated in blocking solution containing 2% normal horse serum, 1% BSA, 0.1% Triton X-100, and 0.05% Tween 20 in PBS. Slides were incubated with a mixture of primary antibodies (RIP3/p-MLKL or BNIP3/CAS3) at the appropriate dilutions in PBS containing 1% BSA for 24 h at 4°C, and fluorescein isothiocyanate (FITC)-conjugated goat anti-rabbit IgG and rhodamine-conjugated goat anti-mouse were used as secondary antibodies (1:500). The nuclei were stained with DAPI and examined under virtual microscopy (BX51 Dot Slide; Olympus, Tokyo, Japan).

### Annexin V-PI Flow-Cytometric Analysis

Apoptotic or necrotic H9c2 cells were detected using Annexin V and PI staining (BD Biosciences, Franklin Lakes, NJ, USA). Annexin V and PI staining were performed according to the manufacturer’s instructions. After staining for 15 min, cells were analyzed by flow cytometry (BD ACCURI C6 cytometer, BD Biosciences). Annexin V−/PI+ cells indicated cells undergoing necrotic cell death. Annexin V+/PI− cells indicated cells undergoing the early stages of apoptosis. Double-positive cells indicated necrotic cell death and the late stages of apoptosis.

### Cell Viability Assay

For the cell viability analysis, cells plated or transfected with miRNAs were exposed to hypoxic conditions for 12 h. Then, Cell Counting Kit-8 reagent (CCK-8, Dogen, Seoul, Korea) was added to each well to a final concentration of 0.5 mg/mL, and the cells were incubated for 2 h. The absorbance at 450 nm was measured using a microplate reader (Thermo Fisher Scientific).

### Luciferase Assay

The whole 3′ UTRs of the target genes regulated by miR-105 were cloned into the pmirGLO vector (Promega). The linker has two different enzyme sites at the 5′ and 3′ ends of the whole 3′ UTR: Xho1-RIP3-Spel and Xho1-BNIP3 Xba1. “Empty plasmid vector” or “plasmid with linker” and miR-105 or NC miRNA were co-transfected into HeLa cells using Lipofectamine LTX with PLUS Reagent (Invitrogen). After 24 h of incubation, luciferase activity was measured using a dual luciferase assay kit (Promega) following the manufacturer’s instructions. Renilla luciferase (Promega) was used to normalize the cell number and transfection efficiency.

### Evaluation of Cardiac Function

For invasive hemodynamics, left ventricular catheterization was performed after 4 weeks post-MI. A Millar Mikro-Tip 2F pressure transducer (model no. SPR-838; Millar Instruments, USA) was introduced into the left ventricle via the right carotid artery (closed chest surgery) under tiletamine/zolazepam (Zoletile, 20 mg/kg) and xylazine (5 mg/kg) anesthesia. Ventricular pressure and real-time volume loops were recorded, and all data were analyzed using Labchart v8.1.5 software (Millar).

### Statistical Analysis

All quantified data are the averages of at least triplicate samples. The error bars represent the SD of the mean. Statistical significance was determined by Student’s t test, and p values ˂ 0.05 were considered significant.

## Author Contributions

S.S., J.-W.C., S.W.K., and K.-C.H. wrote the paper and designed the study. G.H., S. Lim, and S. Lee conceived the study and participated in the design of the study. H.M., J.-H.P., J.L., and H.-H.S. performed the experiments. S.W.K. and K.-C.H. coordinated the work and modified the final manuscript. All authors read and approved the final manuscript.

## Conflicts of Interest

The authors declare no conflict of interest.

## Supplemental Information

Document S1. Figure S1 and Table S1

Document S2. Article plus Supplemental Information
