# Supplementary material for: Simultaneous Suppression of Multiple Programmed Cell Death Pathways by miRNA-105 in Cardiac Ischemic Injury
Source: Mol Ther Nucleic Acids. 2019 Jan 10;14:438–49. doi: 10.1016/j.omtn.2018.12.015 (PMC6369328; doi:10.1016/j.omtn.2018.12.015)
Supplement: Document S1. Figure S1 and Table S1 [file mmc1.pdf]

OMTN, Volume *14*

Supplemental Information

Simultaneous Suppression of Multiple Programmed Cell Death Pathways by miRNA-105 in Cardiac Ischemic Injury

Sunhye Shin, Jung-Won Choi, Hanbyeol Moon, Chang Youn Lee, Jun-Hee Park, Jiyun Lee, Hyang-Hee Seo, Gyoonhee Han, Soyeon Lim, Seahyoung Lee, Sang Woo Kim, and Ki-Chul Hwang

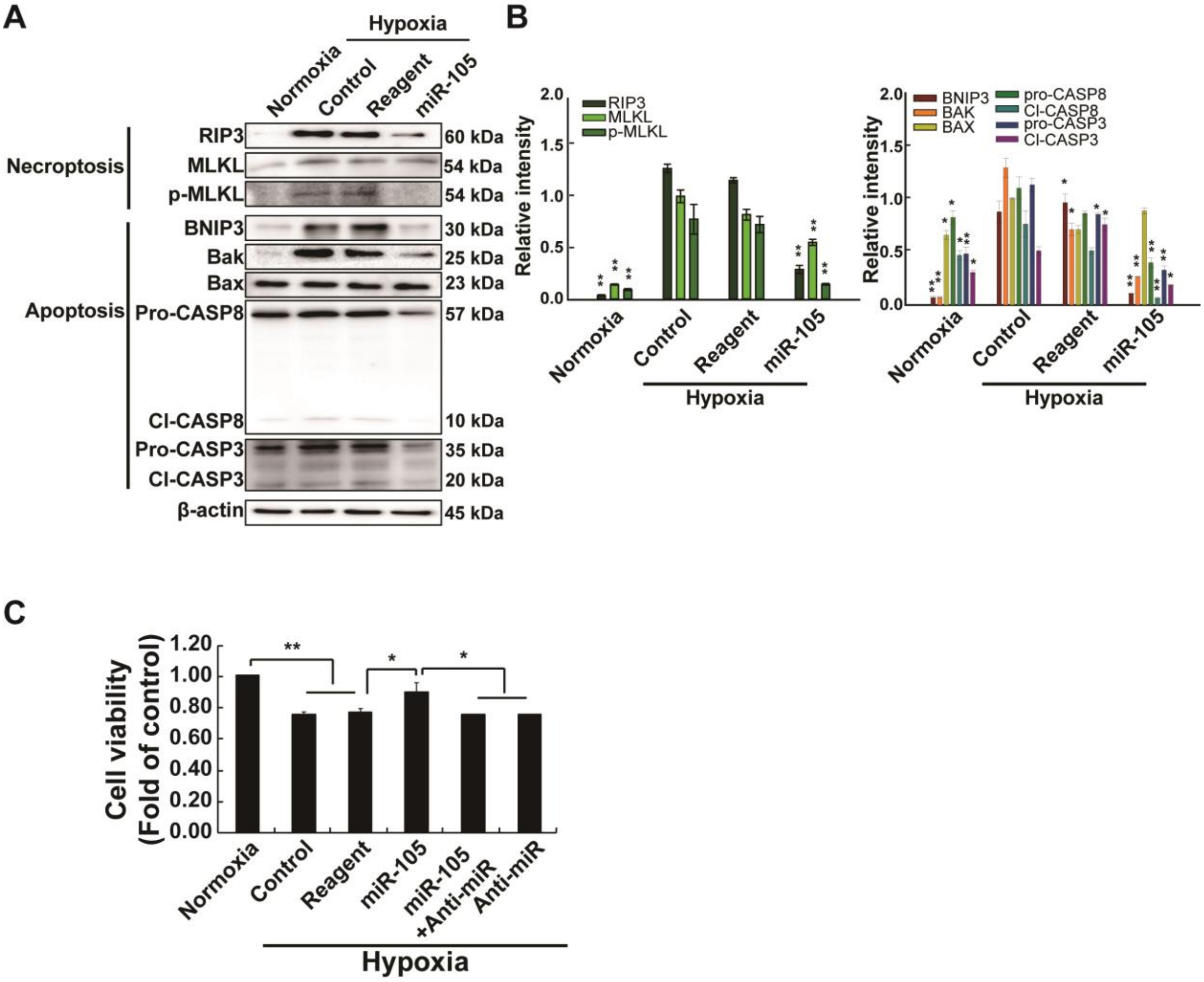

Supplementary Figure S1. Simultaneous suppression of necroptotic and apoptotic cell death by miR-105 transfection in hypoxia-stimulated primary cardiomyocytes. (A) Representative western blot bands showing apoptosis and necroptosis markers. (*n*=4) (B) Band intensities of apoptosis and necroptosis markers. The values given were normalized to the band intensity of β-actin as an internal control. (**p*<0.05, ***p*<0.01, *n*=3). (C) Effects on cell viability by the inhibitor and anti-miR-105. (*n*=3).

Supplementary Table S1. Details for antibodies and primers

Antibody	Cat. No.	Company	Dilutions	
			For WB	For IF
BAK	12105S	Cell Signaling Technology	1:1000	
BAX	ab32503	Abcam	1:1000	
BNIP3	ab10433	Abcam	1:1000	1:100
Caspase-3	Ab1899	MERCK Millipore	1:500	1:100
Caspase-8	9746S	Cell Signaling Technology	1:500	
p-MLKL	91689	Cell Signaling Technology	1:1000	1:100
MLKL	14993	Cell Signaling Technology	1:1000	
RIP3	sc-374639	Santa Cruz Biotechnology	1:500	1:100
β-actin	ab8227	Abcam	1:2000	
GAPDH	sc-47724	Santa Cruz Biotechnology	1:2000	
**Genes**	**Primer sequence (5'-3')**			
Bnip3	Sense strand: GCAGTTGTGTTACGCCTTTATC<br>Antisense strand: GGAGGACGCCTGATTTAACA			
Rip3	Sense strand: GCTGCTGCTTCCAAGGTAAA<br>Antisense strand: GCGGTCCAGCATTTCATAAAC			
Gapdh	Sense strand: GAAAGCCTGCCGGTGACTAA<br>Antisense strand: AGGAAAAGCATCACCCGGAG			
miR-105 (Taqman)	CAAGUGCUCAGAUGUCUGUGGU			
miR-224-3p (Taqman)	AAAUGGUGCCCUAGUGACUACA			
miR-291a-5p (Taqman)	CAUCAAAGUGGAGGCCCUCUCU			
